# Impact of Altitude on Power Output during Cycling Stage Racing

**DOI:** 10.1371/journal.pone.0143028

**Published:** 2015-12-02

**Authors:** Laura A Garvican-Lewis, Bradley Clark, David T. Martin, Yorck Olaf Schumacher, Warren McDonald, Brian Stephens, Fuhai Ma, Kevin G. Thompson, Christopher J. Gore, Paolo Menaspà

**Affiliations:** 1 Research Institute for Sport and Exercise, University of Canberra, Canberra, Australia; 2 Physiology, Australian Institute of Sport, Canberra, Australia; 3 Aspetar Orthopaedic and Sports Medicine Hospital, Doha, Qatar; 4 Cycling Australia, Adelaide, Australia; 5 Qinghai Institute of Sport Science, Duoba, China; 6 Exercise Physiology Laboratory, Flinders University, Adelaide, Australia; 7 Edith Cowan University, Perth, Australia; Hellas, GREECE

## Abstract

**Purpose:**

The purpose of this study was to quantify the effects of moderate-high altitude on power output, cadence, speed and heart rate during a multi-day cycling tour.

**Methods:**

Power output, heart rate, speed and cadence were collected from elite male road cyclists during maximal efforts of 5, 15, 30, 60, 240 and 600 s. The efforts were completed in a laboratory power-profile assessment, and spontaneously during a cycling race simulation near sea-level and an international cycling race at moderate-high altitude. Matched data from the laboratory power-profile and the highest maximal mean power output (MMP) and corresponding speed and heart rate recorded during the cycling race simulation and cycling race at moderate-high altitude were compared using paired t-tests. Additionally, all MMP and corresponding speeds and heart rates were binned per 1000m (<1000m, 1000–2000, 2000–3000 and >3000m) according to the average altitude of each ride. Mixed linear modelling was used to compare cycling performance data from each altitude bin.

**Results:**

Power output was similar between the laboratory power-profile and the race simulation, however MMPs for 5–600 s and 15, 60, 240 and 600 s were lower (*p* ≤ 0.005) during the race at altitude compared with the laboratory power-profile and race simulation, respectively. Furthermore, peak power output and all MMPs were lower (≥ 11.7%, *p* ≤ 0.001) while racing >3000 m compared with rides completed near sea-level. However, speed associated with MMP 60 and 240 s was greater (*p* < 0.001) during racing at moderate-high altitude compared with the race simulation near sea-level.

**Conclusion:**

A reduction in oxygen availability as altitude increases leads to attenuation of cycling power output during competition. Decrement in cycling power output at altitude does not seem to affect speed which tended to be greater at higher altitudes.

## Introduction

Early research has described the performance characteristics of historic competitive cycling events that predominantly take place at low altitudes (< 1000 m). Ebert, et al. [1] determined that cyclists spend most time (~80%) at low to moderate power outputs (2–4.9 W^.^kg^-1^) and least time at highest power outputs (>8 W^.^kg^-1^) during flat, hilly and criterium races. Furthermore, Ebert, et al. [1] reported that for all race types, there are numerous (20–70), short duration bursts (3–30 s) at power greater than the power output associated with maximal oxygen uptake. Others have described cycling events by reporting the typical heart rate response to cycling competition. Although the heart rate response may vary depending on the type and terrain of an event, mean heart rates for competitive road cycling races are ~135 beats per minute (bpm), with higher values (>140 bpm) for time trials or hilly events [2–5]. Cycling cadence and speed, like heart rate, are largely affected by the type and terrain of a race. Generally slowest speeds and cadences are reported during hilly events while fastest speeds and cadences are reported during flat races and time trials [1–3].

In recent years, road cycling events sanctioned by the Union Cycliste Internationale (UCI), have spread globally and are more frequently occurring, either partly or entirely, in moderate-high altitude (2000–5000 m) environments (e.g. Tour of Qinghai Lake, Tour of Utah, stages of Grand Tours). As most famously observed at the 1968 Olympic Games in Mexico City, competition at altitude has consequences for both speed and endurance, with lower air density having a positive impact on speed over short distances, whilst endurance performance is impaired [6]. The change to inspired partial pressure of oxygen at higher altitudes presents a number of challenges to underlying physiological processes that determine or regulate cycling performance. Laboratory based investigations have reported that cycling power output for short to long intervals is attenuated during acute exposure to normobaric and hypobaric hypoxic conditions [7–9]. Reductions in power output during laboratory cycling are likely the result of a decrease in the oxygenation of muscular and cerebral tissue leading to perturbations to working muscle metabolism, central neural drive and pacing regulation [7–10]. Such changes are likely to be reflected by changes in the performance characteristics of real world cycling competitions occurring in similar conditions. Others have reported changes in activity distribution and performance indices during team sports at moderate-high altitude [11–13]. Alternatively, it is possible the reduced air density at altitude results in faster cycling speeds for the same power output, resulting in faster cycling races than at sea level [14–16]. However, to our knowledge no articles exist describing the effects of moderate-high altitude on the performance of cyclists *during* competitive road events.

Therefore, while the performance characteristics of cycling competitions that take place at relatively low altitudes (<1000 m) have been well described, the effects of moderate-high altitude on competitive cycling performance remain largely unknown. Consequently, the purpose of this study was to quantify the acute effects of racing at moderate-high altitude on power output, speed and heart rate compared with sea level efforts in a group of non-acclimatized, low altitude natives. We hypothesized that cycle racing at moderate-high altitude would result in a decrement in mean maximal power (MMP) output, particularly for time durations greater than one minute. Secondly, we hypothesized that cycling speed would be maintained at high altitude due to the reduction in air density.

## Methods

### Study Design

Ride data of elite male cyclists (Table 1) were collected during a seven-day cycling race simulation near sea level (n = 12) and during a 14-day cycling stage race at moderate to high altitude (n = 14) [17]. Cyclists who completed the seven-day race simulation also completed a power-profile test in controlled laboratory conditions, near sea level (n = 12). The study was approved by the Australian Institute of Sport Human Ethics Committee and the Qinghai Institute of Sport Science Ethics Committee. All cyclists provided written informed consent before participating.

**Table 1:** Anthropometric and physiological characteristics and finishing position of cyclists who completed the Australian based race simulation and the Tour of Qinghai Lake (mean ± SD).

	Race Simulation (Australia)	Tour of Qinghai Lake (China)
**n**	12	14
**Height (cm)**	179 ± 4	179 ± 4
**Body mass (kg)**	69.1 ± 5.2	69.7 ± 5.0
**Age (y)**	24.3 ± 2.3	24.6 ± 3.3
**VO**_**2**_ **max (ml·kg·min** ^**-1**^ **)**	74.5 ± 4.9	75.6 ± 4.5
**Training history (y)**	7 ± 3	8 ± 4
**Finishers (n)**	12	9
**Finishing rank**	/	82 ± 24

### Power-Profile Test

The power-profile test [18] was completed by all cyclists involved in the simulated tour (n = 12). The test was conducted at ~600m altitude in controlled laboratory conditions at the Australian Institute of Sport, Canberra using a custom built wind-braked ergometer fitted with a dynamically calibrated scientific eight strain gauge SRM power meter (SRM Training System, Schoberer Rad Messtecknick, Germany), with power sampled at 5 Hz. The individual bicycle riding position of each rider was replicated on the ergometer along with their respective pedal system and crank length. Following a self-selected warm up (~15min at 100–200W including 2–3 rolling sprints), the cyclists were asked to produce sequentially, six maximal self-paced efforts (2 x 5, 15, 30, 60, 240 s) from a rolling start with active recovery periods of 55, 175, 225, 330, and 480 s, respectively. Power data were analysed using commercially available software (SRM, Version 6.42.18) after each test. Specifically, peak power (1 s peak), and the maximum mean power (MMP) and heart rate (HR) for 5, 15, 30, 60, and 240 s were identified. In addition, the MMP for 600 s was predicted using the critical power feature of the publically available software (Golden Cheetah, Version 3.0.2). The power profile test has previously been shown to predict cycling road MMP within race situations [18], and is therefore an ecologically valid tool to assess cycling specific power producing capabilities.

### Race Simulation near Sea Level

In the absence of a suitably comparable tour at sea level, twelve cyclists, from two teams, rostered to compete in the 2013 edition of the Tour of Qinghai Lake completed a simulation of the Tour, near sea level (Canberra and surrounds, Australia) one month prior to the race in China. Due to the racing schedules of the riders, it was only possible to simulate the first seven stages of the race (S 1–7). Each “stage” was re-created based on the course description listed on the race organiser’s website, with the aim being to replicate the stage distance and elevation gain as closely as possible (Table 2). The elevation gain was measured with barometric altimeters (SRM PC7 Schoberer Rad Messtecknick, Germany; or Garmin Edge 500, Garmin, USA) which have been previously shown to provide consistent measures of elevation gain [19]. In addition, race scenarios and tactics, including sprint primes and breakaways were recreated with the assistance of coaching staff in an attempt to introduce race intensity to the simulation. Due to injuries sustained in training during the three weeks prior to the Tour of Qinghai Lake, two cyclists were unable to travel to China and were replaced by reserves. Thus, ten cyclists completed both the simulated tour and the Tour of Qinghai Lake.

**Table 2:** Stage categorisation, distance, average altitude and elevation gain for the Australian based race simulation and Tour of Qinghai Lake.

		Race Simulation (Australia)			Tour of Qinghai Lake (China)		
Stage	Stage Type	Distance (km)	Average altitude (m)	Elevation Gain (m)	Distance (km)	Average altitude (m)	Elevation Gain (m)
**1**	FLAT	120	585	790	138	2265	362
**2**	MTN	177	675	1221	151	2633	1726
**3**	HILLY	147	346	2448	148	3073	1928
**4**	MTN	207	673	1651	227	3325	1488
**5**	HILLY	188	681	1370	203	3287	539
**6**	MTN	164	660	1905	205	3491	1979
**7**	MTN	87	620	1013	82	3055	1650
**8**	HILLY				201	2607	1325
	REST						
**9**	FLAT				117	1482	201
**10**	HILLY				191	1731	935
**11**	FLAT				120	1236	391
**12**	FLAT				123	1159	86
**13**	FLAT				96	1525	75

Stage type categorised by race organisers: FLAT = flat stage, MTN = mountainous stage, HILLY = hilly and undulating stage; Simulated tour performed by Australian riders 1 month prior to the Tour of Qinghai Lake in Canberra and surrounds, Australia at ~580 m.

### Tour of Qinghai Lake

The 2013 Tour of Qinghai Lake was a sanctioned UCI 2.HC (hors category) race and consisted of 13 stages with one rest day after the first eight stages. The race began in Xining, in the Qinghai Province of China and covered 2002 km before the finish in Lanzhou. The average altitude of the entire race was 2496 m, however the first eight stages occurred at highest altitudes (>2000 m, Tour-H), while the remaining five stages took place at lower altitudes (<2000 m, Tour-L). Stages were classified by race organisers as mountainous (MTN), hilly or flat according to the elevation profile of each individual stage. Each team consisted of seven riders and included UCI Pro Tour, Pro-Continental, Continental and National teams. Five riders from the two teams participating in the study were withdrawn from the race to due to injury, illness or failure to complete a stage within the allocated finishing time.

### Ride data

All ride data were collected using dynamically calibrated SRM (SRM Training System, Schoberer Rad Messtecknick, Germany) or Quarq (Quarq Elsa, Quarq, USA) power meters and data loggers (SRM PC7 Schoberer Rad Messtecknick, Germany or Garmin Edge 500, Garmin USA). Each power meter was calibrated using a dynamic calibration rig as described previously [20]. The zero offset for each power meter was configured using the “automatic” function, as per the manufacturers’ recommendations. Power, speed, cadence, distance and heart rate were collected at 1 Hz for the duration of each ride and logged data were downloaded onto a laptop and analysed using publically available software (Golden Cheetah, version 3.0.2).

### Data Analyses

After removing erroneous data files (n = 13) and accounting for rider withdrawal due to injury, illness or failure to complete a stage within the allocated finishing time (n = 5), a total of 187 rides were available for analysis. The following variables were calculated for each ride 1) total race distance (km), time (min), total elevation gain (TEG) (m) and mean altitude (m); 2) mean power (W), speed (km^.^h^-1^), cadence (revolutions^.^min^-1^—rpm), heart rate (beats^.^min^-1^—bpm); 3) peak power and heart rate 4) MMP for 1, 5, 15, 30, 60, 240 and 600 s (W); 5) mean speed associated with MMP for 5, 15, 30, 60, 240 and 600 s; 6) mean heart rate associated with MMP for 1, 5, 15, 30, 60, 240 and 600 s; 7) percentage of total race time spent in relative power bands of < 1.9, 2.0 to 4.9, 5.0 to 7.9 and >8.0 W^.^kg^-1^ (%) [1]; 8) percentage of total time spent in different cadence bands (0, 1 to 60, 61 to 80, 81 to 100, and > 100 rpm (%) [1].

### Statistical Analyses

The 187 rides were initially categorised into: Race simulation 1–7 (S 1–7), race stages 1–7 (R 1–7), Tour stages 1–8 (Tour-H) and Tour stages 9–13 (Tour-L). Ten cyclists completed the laboratory power-profile test, S 1–7 and R 1–7 (Table 3); thus, their matched data were compared using paired t-tests with Bonferroni correction for multiple comparison to determine differences in peak MMP (single highest MMP achieved by each rider during any ride within the category) and associated heart rates and speeds (S 1–7 and R 1–7 only). The same comparisons were made between Tour-H and Tour-L for the nine cyclists who completed the entire race.

**Table 3:** Identification of the riders (by subject number) who completed each period of data collection.

	Laboratory Power-Profile Assessment	Race Simulation Near Sea-Level (S 1–7)	Cycling Race Stages 1–7 (R 1–7)	Cycling Race Stages 1–8 (Tour-H)	Cycling Race Stages 9–13 (Tour-L)
**Riders**	1, 2, 3, 4, 5, 6, 7, 8, 9, 10, 11, 12	1, 2, 3, 4, 5, 6, 7, 8, 9, 10, 11, 12	1, 2, 3, 4, 5, 6, 7, 8, 9, 10, 13, 14, 15, 16	1, 2, 3, 4, 5, 6, 7, 8, 9, 10, 13, 14, 15, 16	1, 2, 3, 4, 5, 6, 13, 14, 15

To further quantify the effects of altitude on cycling performance, the 187 rides were ‘binned’ according to their mean altitude and linear mixed models were fitted using the “*nlme”* package for the statistical program R (Version 3.1.0; R Foundation for Statistical Consulting). Subsequently, altitude was considered a factor of four levels; near sea-level (race simulation and the reference level for altitude), 1000–2000 m, 2000–3000 m and >3000 m. Race characteristics (power, speed, cadence and heart rate), all MMP for 1–600 s and the proportion of race time spent in different power output and cadence bands were fitted as response variables, with altitude and cyclist included as the fixed and random effects respectively. To support interpretation, estimates of the fixed effects, obtained from the log scale, have been converted to a percentage change in each respective response variable and displayed with their 95% confidence intervals. All other variables are displayed as mean ± standard deviation (SD) unless otherwise stated and alpha was set at *p ≤* 0.05.

## Results

### Race Characteristics

Table 4 outlines the general characteristics for rides completed during the race simulation and during the Tour of Qinghai Lake binned according to altitude. Mean power for rides completed during S 1–7 ranged from 186 to 213 W while mean power during R 1–7 ranged from 147 to 194 W. Mean heart rate ranged from 123 to 139 bpm during S 1–7 and was similar R 1–7 (121–142 bpm), while mean cadence ranged from 78 to 91 rpm during S 1–7, and like heart rate, was similar during R 1–7 (80–89 rpm). Conversely, mean speed tended to be lower during the S 1–7 (29.3–28.7 km^.^h^-1^) compared with R 1–7 (28.8–44.1 km^.^h^-1^).

**Table 4:** Distance, altitude, elevation gain, race time, and performance characteristics for rides completed in each altitude bin (mean ± SD).

	Race Simulation Near Sea-Level	1000–2000 m	2000–3000 m	>3000 m
**Rides (n)**	74	36	28	49
** FLAT races (n)**	7	29	10	0
** HILLY races (n)**	22	7	8	21
** MTN races (n)**	43	0	10	28
**Distance (km)**	157.5 ± 38.3	127.1 ± 34.1* ^#^ ^†^	154.1 ± 32.7	172.7 ± 49.5
**Mean Altitude (m)**	604 ± 118^‡^ ^#^ ^†^	1415 ± 211* ^#^ ^†^	2494 ± 174* ^‡^ ^†^	3246 ± 166^‡^ ^#^ *
**Total Elevation Gain (m)**	1544 ± 539	376 ± 329* ^#^ ^†^	1144 ± 645* ^†^	1522 ± 536
**Mean Race Time (min)**	280.0 ± 64.2	164.6 ± 47.2* ^#^ ^†^	219.3 ± 44.3* ^‡^ ^†^	283.9 ± 64.5
**Mean Power (W)**	205 ± 24	200 ± 30	173 ± 21* ^‡^	175 ± 24* ^‡^
**Mean Cadence (rpm)**	83 ± 5^‡^ ^#^	88 ± 4	86 ± 3	82 ± 4^‡^ ^#^
**Mean Speed (km·h** ^**-1**^ **)**	33.8 ± 3.4^‡^ ^#^	46.6 ± 2.3	42.4 ± 3.2	36.0 ± 4.1* ^‡^ ^#^
**Mean Heart Rate (bpm)**	132 ± 10	134 ± 11	133 ± 11	132 ± 11
**Max Heart Rate (bpm)**	176 ± 10	179 ± 8	176 ± 15	170 ± 8* ^‡^ ^#^

*Significantly different (P < 0.05) from Race Simulation

‡Significantly different from 1000–2000 m

^#^Significantly different from 2000–3000 m

†Significantly different from >3000 m.

### Power Output

**Fig 1:**
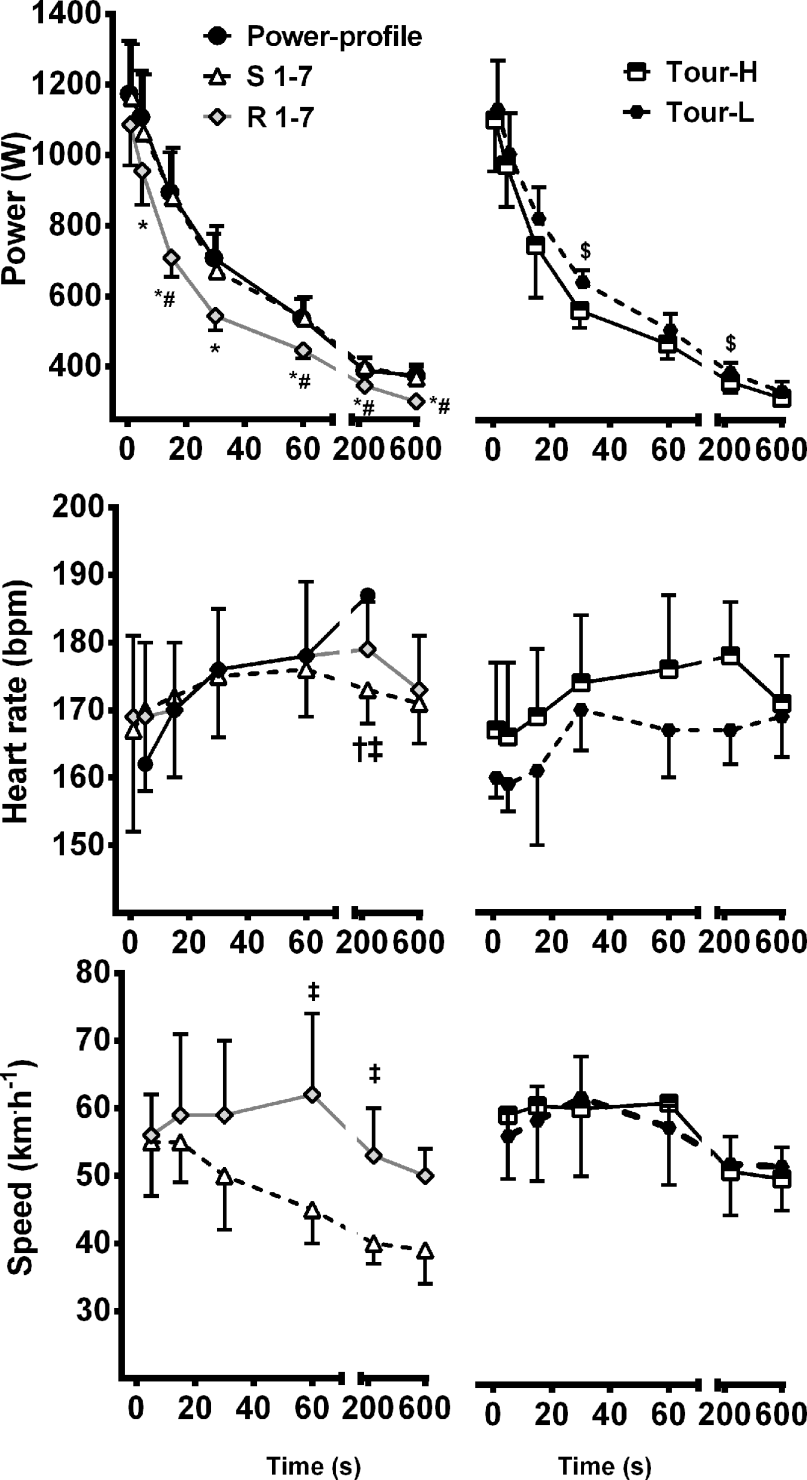
Time-Power, time-speed and time-heart rate relationship. Left panels compare the lab power-profile test with the race simulation in Canberra (S 1–7) and the first 7 stages of the race in China (R 1–7); n = 10. Right panels compare Tour-H (altitude > 2000 m) with Tour-L (altitude < 2000 m); n = 9. *Power-profile significantly higher than R 1–7 (P < 0.05); # S 1–7 significantly higher than R 1–7; † power-profile significantly higher than S 1–7; ‡ R 1–7 significantly higher than S 1–7; $ Tour-L significantly higher than Tour-H.

There were no significant differences between MMP measured during the laboratory power-profile test and peak MMP measured during S 1–7 (Fig 1). However, MMP 5–600 s were significantly lower (*p* ≤ 0.003) during R 1–7 compared with the laboratory power-profile and MMP 15, 60, 240 and 600 s were significantly lower (*p* ≤ 0.004) during R 1–7 compared with the S 1–7. Generally, MMP’s were similar for Tour-H compared with Tour-L, however MMP 30 and 240 s were significantly higher during Tour-L when compared to Tour-H.

**Fig 2:**
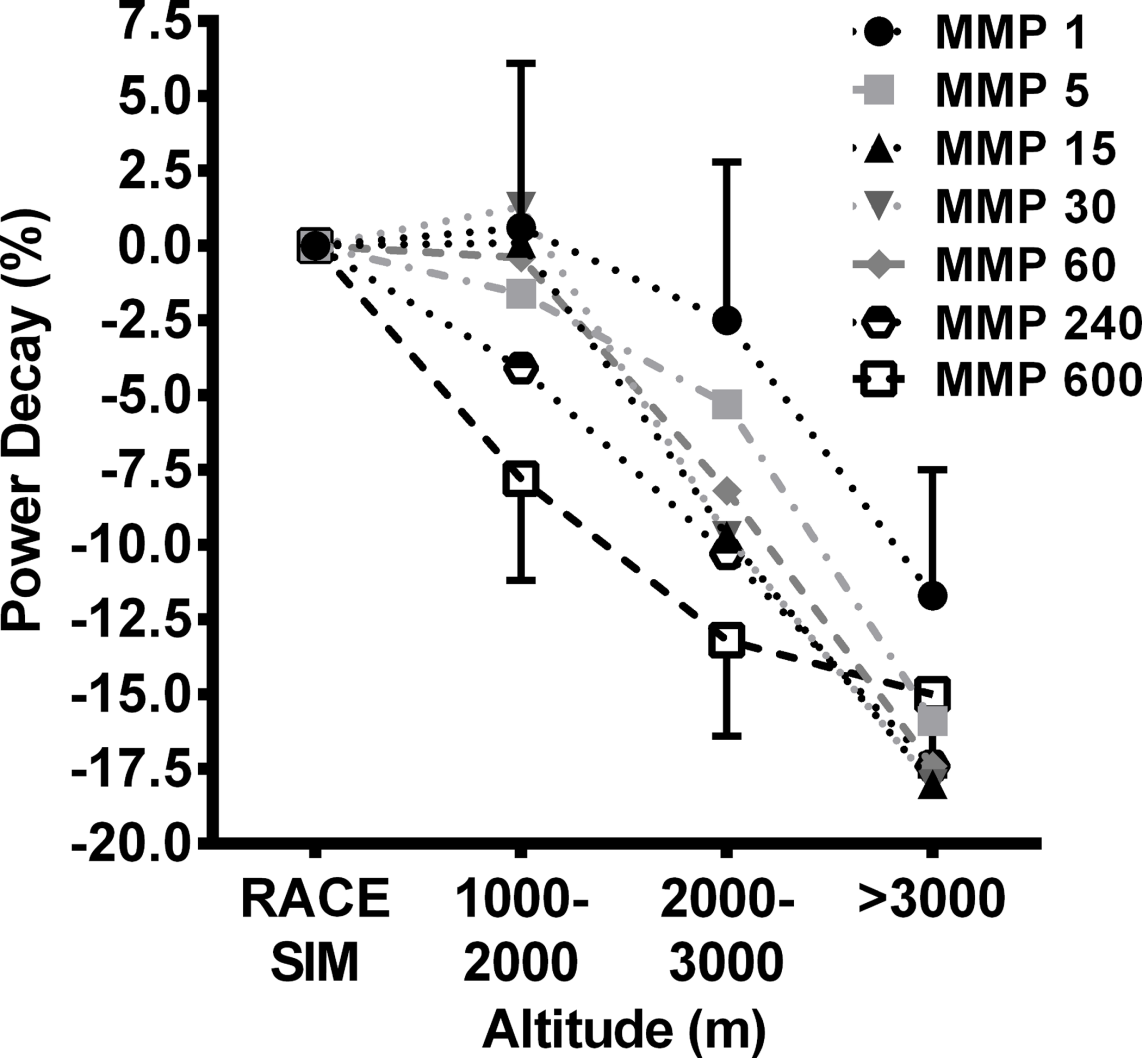
Rate of decline for specific mean maximal power as altitude increases. RACE SIM = Race simulation completed near sea-level in Australia.

When comparing rides completed in different altitude bins, MMP for 1 and 5 s were similar for races completed below 3000 m altitude (Fig 2). However, during rides above 3000 m, MMP 1 s and MMP 5 s were 11.7% (7.5–15.7%, *p* < 0.001; mean and 95% confidence interval [CI]) and 15.9% (10.7–20.8%, *p* < 0.001) lower, respectively, than rides completed near sea-level. Maximal mean powers for 15, 30 and 60 s were substantially lower during rides completed at 2000–3000 m altitude compared with rides near sea-level and declined further during rides completed above 3000 m. Maximal mean power 240 and 600 s were lower by 4.1% (1.0–7.1%, *p* = 0.01) and 7.8% (4.4–11.2%, *p* < 0.001), respectively, at 1000–2000 m compared with near sea-level and, like shorter duration MMP’s declined further at higher altitudes; being 17.4% (15.1–19.6%, *p* < 0.001) and 15.0% (12.3–17.7%, *p* < 0.001) lower at >3000 m compared with rides near sea-level.

**Fig 3:**
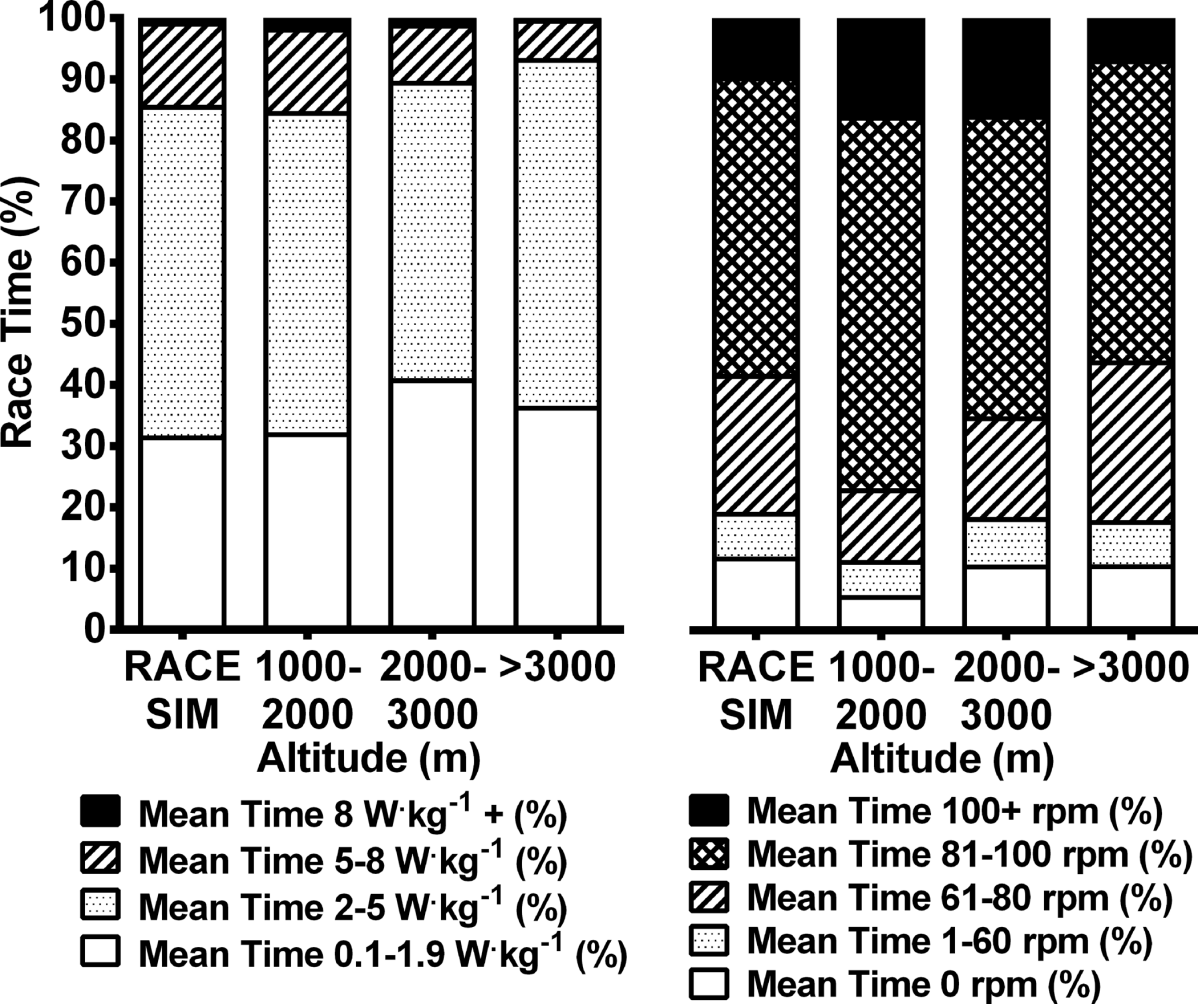
Proportion of overall race time spent in relative power (a) and cadence bins (b). RACE SIM = Race simulation completed near sea-level in Australia.

Overall mean power was similar for all rides completed below 2000 m altitude (Table 4). However, for rides completed at 2000–3000 m and above 3000 m, mean power was 12.4% (8.1–16.6%, *p* < 0.001) and 12.3% (8.6–15.9%, *p* < 0.001) lower, respectively, than rides completed below near sea-level. Differences in mean power are reflected by the relative time spent within different power bins (Fig 3) recorded for each altitude condition. Cyclists spent a greater proportion of overall riding time below 2 W^.^kg^-1^ during rides completed at 2000–3000 m (39.6%, 35.6–44.0%) and above 3000 m (34.4%, 31.5–37.7%) compared with rides at 1000–2000 m (30.2%, 27.3–33.4%, *p* ≤ 0.01) and near sea-level (31%, 29.0–33.1%, P ≤ 0.02). Conversely, time spent in the 5–8 W^.^kg^-1^ power bin was higher during rides near sea-level (12.5%, 11.3–14.0%) and 1000–2000 m (13.2%, 11.7–14.8%) when compared to 2000–3000 m (9.1%, 8.0–10.2%, *p* < 0.001) and >3000 m (6.0%, 5.4–6.7%, *p* < 0.001). For all conditions, time spent in the 2–5 W^.^kg^-1^ and 8+ W^.^kg^-1^ power bins was similar with the exception of rides completed at 1000–2000 m during which cyclists spent slightly more time above 8 W^.^kg^-1^ (2.0%, 1.6–2.6%, *p* < 0.001) compared with rides at other altitudes.

### Heart Rate

The heart rates associated with MMP were similar between the power-profile, S 1–7 and R 1–7 with the exception of 240 s (Fig 1), which was significantly lower during S 1–7 compared with the power-profile (*p* = 0.001) and Tour stages 1–7 (*p* = 0.002). There were no significant differences in any MMP associated heart rates between Tour-H and Tour-L (Fig 1). When comparing rides within each specific altitude bin, mean heart rate was similar for rides in all altitude conditions (Table 4). However, peak heart rate was 2.7–5.0% (*p* ≤ 0.02) lower during rides completed above 3000 m compared with other altitude conditions.

### Speed

Speeds associated with MMP were similar between S 1–7 and R 1–7 with the exception of MMP 60 and 240 s which were significantly higher during R 1–7 (*p* = 0.003 and 0.002, respectively). Conversely, speeds for all MMPs were similar between Tour-H and Tour-L. Mean speed was substantially lower (7.3–39%, *p* ≤ 0.012) during rides completed below near sea-level compared with rides in all other altitude conditions (Table 4).

### Cadence

Mean cadence was similar for rides completed near sea-level and above 3000 m; however for rides at 1000–2000 m and 2000–3000 m cadence was ~7.3% and ~4.6% (*p* < 0.001) higher than rides completed at either lowest or highest altitudes (Table 4). In all altitude conditions, cyclists spent the greatest proportion of ride time at 80–100 rpm (47.3–58.8%); however for rides at 1000–2000 m and 2000–3000 m, cyclists spent a greater portion of ride time at cadences above 100 rpm (Fig 3).

## Discussion

To our knowledge, this is the first study describing the effects of moderate-high altitude on performance during an international multi-day road cycling competition. The main finding of the present study was a significant reduction in the maximal mean power outputs of sea-level natives during cycling stage racing at moderate-high altitudes compared with laboratory cycling assessment and a race simulation near sea-level. Additionally, power output, heart rate and cadence data indicate that the simulated race situations provided a valid representation of the cycling competition.

### Validity of the race simulation

In lieu of a suitably comparable cycling event near sea level, a race simulation was used to examine the performance of sea-level natives during low altitude cycle racing. A somewhat crude comparison of mean power output, speed, heart rate and the relative time spent within different power zones observed during the race simulation and reported by other investigations of road cycling competitions [1–5] indicates that the race simulation was largely effective in mimicking the physiological demands of real competition. However, more sophisticated evaluation of MMP recorded for discrete time periods further indicates the simulation was a valid example of sea-level racing. Specifically, there were no differences between power measured during a laboratory power-profile test, which is highly predictive of sea-level performance capability [18], and peak MMP’s recorded across the race simulation. Additionally, the heart rates associated with simulation MMP and laboratory power-profile were similar, with the exception of 240 s which was higher during the laboratory test. Therefore, given the broad similarities between the race simulation, laboratory power-profile and race profiles from earlier investigations, the race simulation appears to provide a valid representation of the physiological and performance characteristics of cycling competition near sea level, and hence provides a reference point for comparison to competition at altitude in the present study.

### Effect of altitude on cycle racing power output

Exercise capacity was perturbed during moderate-high altitude cycling stage racing, manifesting as a reduction in peak MMP for 5–600 s and 15, 60, 240 and 600 s when compared to laboratory power-profile assessment and the race simulation near sea-level, respectively. The reduction in longer duration MMP (≥ 240 s) observed in the current study is likely the result of impairment of the aerobic energy system at higher altitudes. An increase in altitude is associated with a reduction in arterial oxygen availability and oxygen delivery during exercise [21–23] which leads to a linear reduction in maximal oxygen uptake as altitude increases, particularly in highly trained endurance athletes [21, 24, 25]. Indeed, further analysis using all MMPs (i.e. not just the peak) recorded throughout the race simulation and Tour of Qinghai Lake revealed a decline in MMP for 240 and 600 s of ~6% per 1000 m above sea-level, confirming the results of earlier laboratory analyses [8, 21, 25]. However, as one of a multitude of factors that determine performance [26], it is unlikely a decline in oxygen uptake explains all changes observed in the present study. Alterations in the ventilatory response to exercise, including an increase in ventilation [21] and the ventilatory equivalents for O_2_ and CO_2_ [22], have also been reported in hypoxia. Additionally, Valli, et al. [27] suggested the hyperventilatory response to hypoxia could create a diversion of blood flow to the inspiratory muscles. The overall sum of the ventilatory response to hypoxia is therefore likely to increase the energy and O_2_ cost of inspiration and inhibit exercise performance at high altitude.

An interesting and novel finding of the present study was a decline in short duration and high intensity cycling (MMP ≤60 s) for which the contribution of oxidative energetic pathways is low [28]. In contrast, previous investigations reported no decline in sprint cycling performance in hypoxia when performed as an isolated 30 s effort [29] or as the first in a sequence of 10 s efforts [30]. However, the MMPs recorded in the current study did not occur in isolation but more likely after earlier epochs of variable intensity cycling as necessitated by tactics and the actions of other racers. In this regard, Smith and Billaut [30] indicated a greater amount work was completed in normoxia compared with hypoxia when sprints were repeated multiple times and therefore with an existing load from previous efforts. Interestingly, Smith and Billaut [30] pointed to a marked increase in cerebral deoxygenation as being the predominant cause of reduced sprint performance in hypoxia. Further, research indicates central neural drive is inhibited during intense exercise in hypoxia as a result of cerebral deoxygenation [9, 10, 31] a reduction in cerebral blood flow [9] and faster rates of peripheral muscle fatigue [7]. It is likely the sprint performances of riders in the current study were limited by similar perturbations in central neural drive, reflecting a reduction in global exercise capacity and not just exercise performance that has a high aerobic energetic contribution.

Reductions in MMPs were reflected by a change in the overall activity profile of cycle racing at higher altitudes. Like previous investigations of team sports [11, 12] we observed a decrease in the proportion of time spent at higher exercise intensities. Additionally, there was a concomitant increase in the time spent at lower exercise intensities and mean power output was ~12% lower during races completed above altitudes of 2000 m. Similar to previous investigations with team sport athletes, the capacity for high intensity efforts was not preserved even with the shift to lower overall exercise intensity.

### Effect of altitude on heart rate, speed and cadence

Cycling at higher altitude presents an interesting paradox of increased speed despite inhibition of underlying physiological processes and exercise capacity. Various models indicate the optimal altitude for short to medium duration (5 s–1 hr) cycling performance is 2500–4000 m [6, 16, 32, 33] and as a result many cyclists select moderate-high altitude locations for world record attempts. In the current study, although power output decreased at higher altitudes, there was no negative effect on speed and in most instances speed was higher during the Tour of Qinghai Lake compared with the race simulation near sea level. However, it is possible the higher speeds recorded during racing were due to the larger size of the cycling peloton (in contrast to our race simulation) which offers aerodynamic advantages [34] additional to reduced air density at higher altitudes, as well as the circuit nature of some of the stages [1].

Given previous investigations have reported higher heart rates during sub-maximal exercise in hypoxia [8, 21], it could be expected to observe higher mean heart rates during racing at moderate-high altitude. As mean heart rate was similar between rides within each altitude bin, it could therefore be suggested that hypoxia has little effect on heart rates during sub-maximal exercise. However, as mean power was lower for rides completed above 2000 m, it would appear that sub-maximal heart rate was higher in response to hypoxia during cycling racing. In contrast, peak heart rate tended to be lower particularly during racing above 3000 m altitude. Previous research has also reported a reduction in peak heart rate as altitude increases [23, 27] and the difference in peak heart rate between sea-level and 3000 m of ~6 bpm supports the earlier finding of Wehrlin and Hallen [21] of a decrease in peak heart rate of ~2 bpm per 1000 m.

Mean cadence tended to be lower for rides completed either near sea-level or >3000 m. However this is more likely an artefact of the hillier nature of rides completed at these altitudes, during which elevation gain was highest, as opposed to an effect of altitude. Similar to previous investigations, cyclists spent the greatest proportion of all races at 80–100 rpm [1]. Therefore it would seem that altitude in this instance has little effect on cycling cadence and cyclists likely adjust gear ratios to match the lower power outputs.

### Limitations

Given the applied nature of the research, the findings outlined above must be treated relative to some limitations. Although careful consideration was given to the design of the race simulation, it was not possible to control for all race scenarios, the size of the respective groups and the actions of competitors during the Tour of Qinghai Lake. Furthermore, given the time demands of participants, it was not possible to simulate all stages at sea-level prior to competition. Nevertheless, this study provides important empirical evidence of the changes in cycling racing performance in moderate to high altitude environments.

### Practical implications

The changes in MMP, mean power and the global activity profile described above indicate an overall decline in exercise performance capacity as altitude increases. Such a decline has important implications for athlete load management particularly given the growing trend towards modelling the response to the training or competition stimuli. Therefore it would seem prudent that scientists, coaches and athletes make changes to the parameters of respective models to reflect an overall decline in performance capacity of ~6% per 1000m above sea-level. Competition organisers could also consider the decline in performance when planning events at high altitudes which are likely to elevate physiological load and adversely affect the welfare of competitors.

## Conclusion

Maximal mean power outputs for 1–600 s were attenuated during cycle racing at moderate and high altitudes compared with a laboratory power assessment and low altitude racing or race simulation. Reductions in MMP’s are likely the result of reduced FiO_2_ associated with high altitude environments and the subsequent decline in oxygen availability at the musculature of the lower limb. However, it is possible short term MMP (1–30 s) is moderated by lower central neural drive as it is unlikely limitations in oxygen availability would limit short duration power output. Finally, with consideration for course design, specific race scenarios and sufficient group sizes, it is possible to simulate a cycling road race with similar power output, heart rate and cadence demands as real race events. Such simulations could serve as a valuable tool in future investigations of performance altering interventions, or to further examine the physiological response to cycling competition.
